# Parietal abdominal pain with lower leg discrepancy: a case report

**DOI:** 10.1186/s13256-024-04489-0

**Published:** 2024-04-12

**Authors:** Agnès Gritli, David Cadavid Ramirez, Pierre Decavel

**Affiliations:** Department of Readaptation, HFR Fribourg Hôpital cantonal, chemin des pensionnats 2-6, 1708 Fribourg, Switzerland

**Keywords:** Parietal abdominal pain, Lower leg discrepancy, Botulinum toxin

## Abstract

**Background:**

This report involves the first publication describing a case of parietal abdominal pain due to lower limb length discrepancy.

**Case presentation:**

A Caucasian male patient in his 50s was referred to our rehabilitation department with chronic abdominal pain that began in childhood. This chronic pain was associated with episodes of acute pain that were partially relieved by grade 3 analgesics. The patient was unable to sit for long periods, had recently lost his job, and was unable to participate in recreational activities with his children. Investigations revealed contracture and hypertrophy of the external oblique muscle and an limb length discrepancy of 3.8 cm (1.5 inches) in the left lower limb. The patient was effectively treated with a heel raise, physiotherapy, intramuscular injection of botulinum toxin, and lidocaine. The patient achieved the therapeutic goals of returning to work, and reducing analgesic use.

**Conclusions:**

Structural misbalances, as may be caused by lower leg discrepancy, may trigger muscular compensations and pain. Complete anamnesis and clinical examination must not be trivialized and may reveal previously ignored information leading to a proper diagnosis.

## Background

### Introduction

The prevalence of limb length discrepancy (LLD), also referred to as leg length discrepancy in the literature, is extremely variable across studies and definitions, ranging from 4% [1] to 95% [2]. However, in otherwise healthy individuals, its prevalence is estimated of 30%. The mean limb discrepancy is 5 mm (0.2 inches) [3]. LLD is associated with scoliosis, lower back pain, hip osteoarthritis, knee pain, ankle joint equinus, and hallux valgus [4].

While numerous studies have investigated the impact of functional scoliosis on trunk balance, this balance also involves the abdominal muscles [5]. Abdominal muscles, by their tone, participate in the containment of the intra-abdominal organs and in body statics. Therefore lower limb length inequality likely impacts the abdominal muscles. However, no case involving these muscles has been reported in the literature to date. Here we report a case of chronic abdominal pain in association with LLD.

## Case presentation

### Patient information

A white Caucasian male patient in his 50s presented with chronic abdominal pain of an undetermined etiology. The pain began during childhood, and it remained consistent until increasing in intensity a decade ago. The patient described the pain as a continuous burning sensation primarily localized on the left flank. Patient rates his pain as 8–9 on the numerical rating scale from 0 to 10 (NRS-11) [6]. Pain increased when the trunk muscles contract. This pain obtained a score of 3 on Douleur Neuropathique diagnostic questionnaire (DN4; cut off of positivity: 4) and negative score based on the Budapest complex regional pain syndrome diagnostic criteria [7].

A detailed anamnesis revealed a surgical history involving the implantation of a subcutaneous neurostimulator in the right flank with a probe ending in the epidural space in Th9–Th10 in 2019, which had no effect on the patient’s abdominal pain. In 2010 a hip benign tumor (osteoid osteoma) was treated by resection and implantation of a prosthesis in 2010. There was no documentation of lower limb length inequality either before or after surgery. The patient also underwent resection of the infra-umbilical abdominal nerves in 2021 based on the hypothesis that he had anterior cutaneous nerve entrapment syndrome. However, this had no conclusive effects. The patient had been prescribed morphine for 15 years; at the time of the evaluation, he was taking 230 mg per day.

The patient was unable to sit for longer than 30 minutes. This along with the intensity of the pain resulted in the patient losing his job in 2020. Furthermore, it restricted his social activities, such as being able to accompany his children to soccer games.

### Clinical findings

**Fig. 1:**
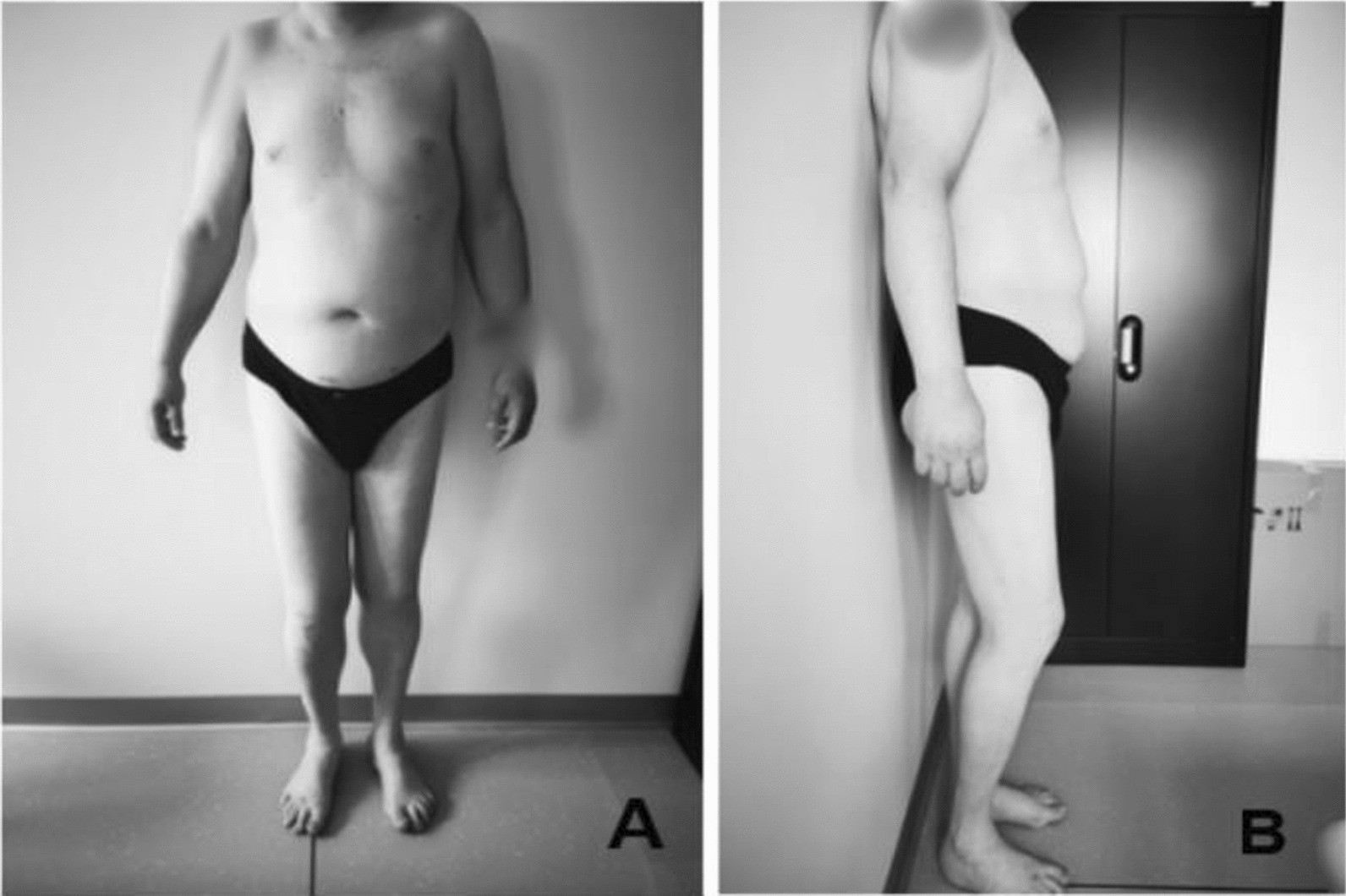
A clinical photograph showing bending of the right knee while standing, with a tilt of the right shoulder on the same side. **A** Front view, **B** side view. Scars are present on the infra-umbilical abdomen but not elsewhere

Clinical examination revealed a visible inequality in the length of the lower limbs, combined with tilting of the pelvis and compensatory rotation of the trunk to the right (Fig. 1). Measurement from the anterior superior iliac spine to the medial malleolus [8] for each extremity revealed a length discrepancy of 4 cm (1.6 inches) in the left lower limb. Standing on blocks with a spirit level [9] placed on both iliac crests confirmed a LLD of 4 cm (1.6 inches).

Analytical examination of hips and knees were symmetric; however, there was a more limited, albeit painless, range of motion on the left side (arthroplasty). No lower limb muscle contracture or loss of extensibility was observed.

The patient’s gait cycle began with initial heel contact on both sides. During the stance phase, the shorter limb exhibited pelvis anteversion, decreased hip flexion, full knee extension, and increased ankle plantar extension. The longer limb exhibited a relative pelvis retroversion and pronounced hip, knee, and ankle planter flexion. During the swing phase, the shorter limb swayed with hip, knee, and ankle plantar extension, while the longer one swayed maintaining a hip, knee, and ankle plantar flexion. This was associated with a posterior tilt of the right shoulder, compensatory rotation of the trunk to the right, and symmetric arm balancing.

The patient’s pain increased following left flank palpation with no guarding. Muscle contracture was palpable under the skin. Active maneuvers lead to increased pain with flexion and a right rotation of the trunk from the sitting position. The sub-umbilical part of the trunk was also painful, with a pain increase following muscle palpation and active flexion of the trunk from the sitting position. The rest of the clinical examination was unremarkable.

### Timeline

**Table:** 

2010	Osteoid osteoma resection and hip prosthesis implantation
2015	Left median branch block from Th10 to L1
2019	Subcutaneous neurostimulator implantation
April 2021	Infra-umbilical abdominal nerves resection
August 2021	Start rehabilitation follow-up
August 2021	Teleroentgenogram
August 2021	Corrective heel rise
August 2022	Abdominal wall ultrasound
January 2022	Abdominal wall muscles electromyography
January 2022	Infiltration of external oblique and rectus abdominis muscle
August 2022–February 2023	Weekly 30-minute long physiotherapy sessions
August 2021	First botulinum toxin-A injections on the external oblique muscle
November 2021	Second botulinum toxin-A injections on the external oblique muscle
January 2022	First injection of lidocaine in the inferior portion of the right abdominal muscles
February 2022	Third botulin toxin-A injection into the right abdominal muscle
February 2022	Third botulin toxin-A into the right abdominal muscle
August 2022	End rehabilitation follow-up

### Diagnostic assessment

The patient provided the equivalent of a decade’s worth of medical investigations, among which numerous imaging examinations were carried out, including abdominal ultrasonography, spine X-rays, abdominal computed tomography (CT) scans, and lumbar magnetic resonance imaging (MRI). None demonstrated abdominal fibrosis or other etiologies that may explain his symptoms; visceral and vascular etiologies were excluded. Biological investigations (hemogram, ionogram, inflammation, and bacteriology) revealed no abnormalities.

**Fig. 2:**
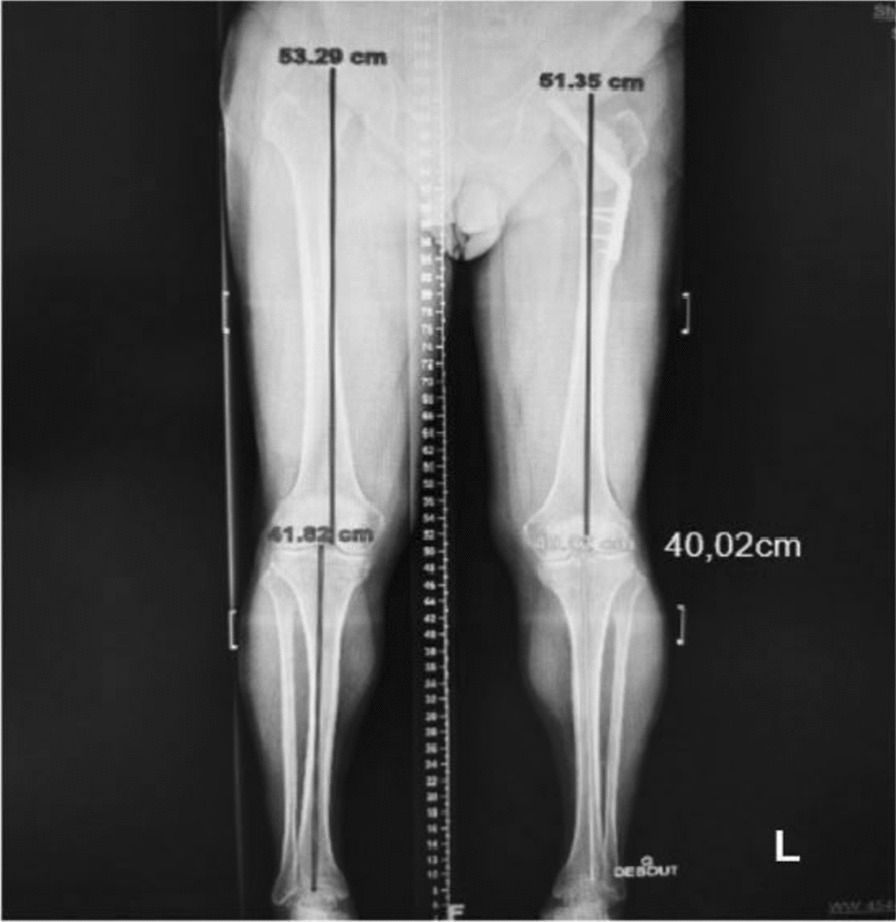
Teleroentgenogram of the lower limbs demonstrating leg length discrepancy, with left femoral and tibial shortening and “gamma nail” in the left femoral head

In this evaluation no novel investigations were performed to exclude other diagnoses, as these prior explorations were deemed sufficient. However, the lower limb discrepancy and abdominal muscle thickness were measured. A teleroentgenogram of the lower limbs was performed, demonstrating asymmetrical limb length, with an estimated shortening of 3.8 cm (1.5 inches) on the left side (Fig. 2).

Following this diagnosis, an ultrasound of the left external oblique (EO) muscle demonstrated a thickening of 2.3 mm (0.04 inches). The thickness of the lateral abdominal muscles was measured at rest on both sides. The patient was placed in the supine position with the hips flexed at 30°.

**Fig. 3:**
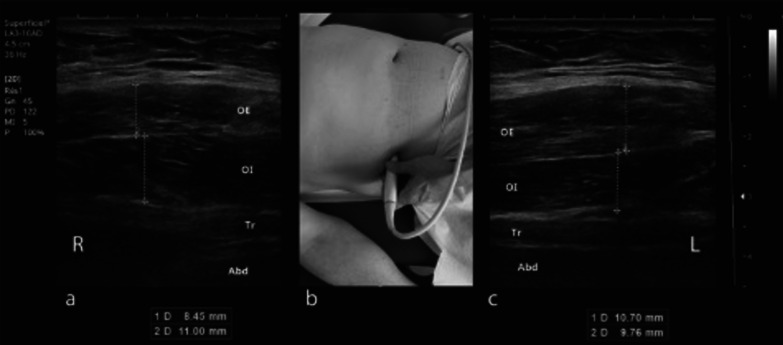
Ultrasound of the right flank (**a**), with thickness measurement of the external oblique (EO) at 8.45 mm and of the internal oblique (IO) at 11 mm. Ultrasound was performed bilaterally placing the probe at shown location (**b**) comparatively on both sides, finding at left (**c**) an EO at 10.7 mm and IO at 9.76 mm. We noticed a difference of 1 mm on the left side and 2.55 mm on the right side (and an inversion of the relative thickness)

The transducer was placed 25 mm antero-medial to the midpoint between the last rib and the ilium on the mid-axillary line, where the fascia margins between the transverse abdominal (TrA), internal oblique (IO). and EO are parallel [10]. The thickness of the abdominal muscles was recorded using HS40 (Samsung Inc, Seoul, KOR) with a 3–16-MHz, 60 mm linear array. Electromyography was performed during the therapeutic procedure using DANTEC CLAVIS (Natus INC, Middletown, WI), which can give an auditory response only (see below), and this confirmed abnormal muscle overactivity. An ultrasound was performed at the lower part of the rectus abdominis (RA) and demonstrated no significant difference in thickness (Fig. 3).

### Differential diagnosis

LLD with a multifactorial etiology was confirmed [that is, both congenital (tibial) and postoperative (after hip osteosynthesis) origins].

Several differential diagnoses were made:

1. Intra-abdominal pain. The patient presented with well-localized abdominal wall pain syndrome with a positive Carnett test. Regarding the patient’s chronic pain, visceral, fibrotic, and vascular etiologies were excluded via ultrasonography and CT.
2. Anterior cutaneous nerve entrapment (ACNES) syndrome. The patient experienced dual pain location. Concerning the right flank pain, the location was higher than the RA. A negative fingertip test was returned for the left anterior abdominal wall. A positive Carnett test was returned. However, this was only for the combined flexion and right rotation of the trunk due to EO muscle contraction; flexion alone did not increase the patient’s pain. Infiltration of anesthetic drugs into the EO muscle significantly decreased the pain. Previous treatments involving multiple infiltrations of ropivacaine with an analgesic, left median branch block from T10 to L1 and the resection of the infra-umbilical abdominal nerves had been unsuccessful in decreasing the patient’s pain. Regional nerve block is an effective method for eliminating a potential diagnosis of ACNES as well as for providing optimal long-lasting pain relief [11–14].
3. The truncal complex regional pain syndrome (CPRD) is a rare diagnosis that was excluded for several reasons. First, the pain was described as a burning and continuous sensation localized to the flanks, that first began during childhood and increased with trunk muscle contractions. Furthermore, there was no surgical history during childhood, and the pain was partially relieved by morphine. Additionally, bilateral muscle contracture was noted during the clinical examination, and the intramuscular administration of botulinum toxin and LLD correction eliminated the pain. As revealed by the clinical examination and the figures, the patient does not present any sensory-motor alteration, edema, or trophic modification.

### Therapeutic intervention

In collaboration with the patient, shared objectives were formulated based on the International Classification of Functioning (ICF) framework [15]. The first step was to treat the LLD to decrease the level of pain using the NRS-11. The improvement in pain was expected to result in the patient’s improved ability to sit, improved capacity to return to work, and ability to enjoy leisure activities with family. These two elements were evaluated subjectively based on what the patient reported.

Treatment initially aimed to correct the LLD, using a corrective heel raise of 2 cm (0.8 inches). No larger heel insoles or further shoe raises were proposed since the literature supports gentle and progressive discrepancy correction [16]. With this correction and further infiltrative treatment, the patient’s symptoms were satisfactorily relieved. Therefore, no additional corrections were needed. Physiotherapy sessions were conducted on a weekly basis for a period of 6 months. Each 30-minute session included deep abdominal musculature reprogramming exercises, stretching and massage of the EO and right IO, and one- and two-leg standing balance exercises. Progressions in movement complexity were added based on body posture.

**Fig. 4:**
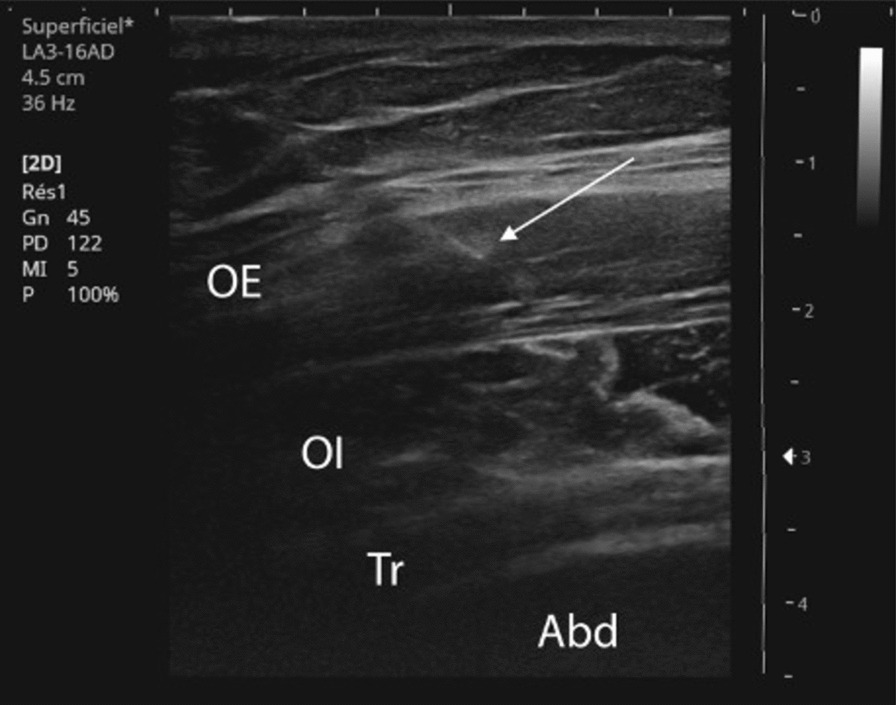
The needle in the external oblique muscle (white arrow). *OI* internal oblique muscle, *Tr* transversal abdominal muscle, *Abd* abdominal cavity, *OE* external oblique muscle

Following this, two rounds of 100 IU of botulinum toxin-A were injected into the EO muscle under ultrasound guidance and electromyographic tracking, twice (Fig. 4). The first injection enabled reduction in analgesic intake, and the second drastically albeit transiently reduced pain (for less than 12 hours). The third treatment involved performing an intramuscular block by injecting 3 mL of lidocaine into the EO, and a further 2 mL on each side in the lower part of the RA under ultrasound guidance and electromyographic tracking.

This procedure resulted in a permanent immediate decrease in the right flank (EO) pain, as well as some reduction in the sub-umbilical trunk (RA).

**Fig. 5:**
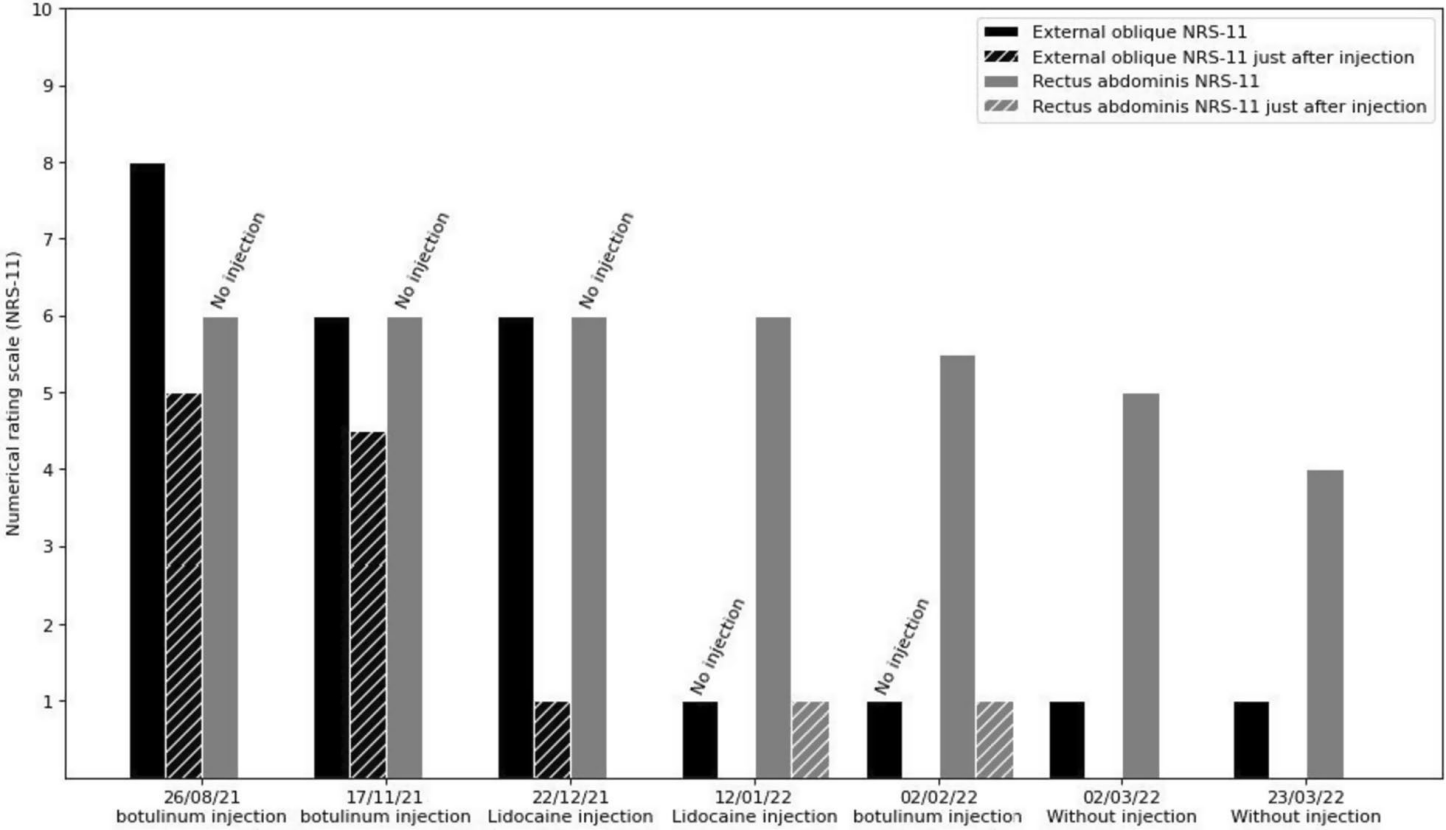
Numerical rating scale-11 evaluation of pain in the oblique external muscle and lower rectus abdominis (RA) over time and after-injection. *NRS-11* numeric rating scale-11, *EO* oblique external muscle, *RA* rectus abdominis

The last treatment involved injecting botulinum toxin-A into the RA under ultrasound guidance and electromyographic tracking. This resulted in a slow and progressive reduction in pain (Fig. 5).

### Follow-up and outcomes

**Fig. 6:**
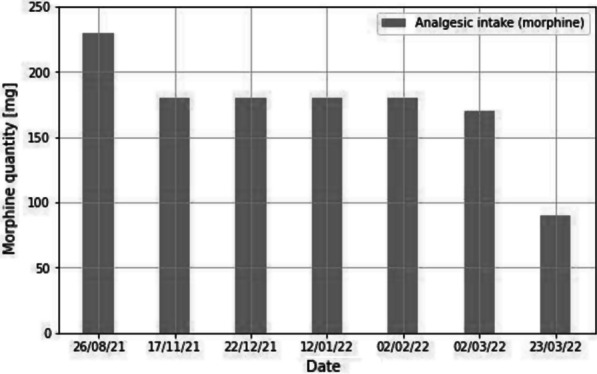
Course of analgesic intake over time

The follow-up period lasted 7 months. The NRS-11 was used to monitor changes in pain and analgesic intake. The numerical results were in line with the patient’s clinical outcome, whereby there was an immediate response to muscle-blocking treatment in terms of a reduction in chronic pain. The patient reported an almost complete elimination of pain after repeated blocks treatments in conjunction with physiotherapy and decreased pharmacologic intake (Fig. 6). No adverse events were reported during the follow-up period.

## Discussion and conclusions

This is the first publication to describe abdominal muscle pain related to LLD. The degree of LLD required to cause such disorders remains a topic of debate. Rannisto *et al* found that LLDs greater than 6 mm (0.3 inches) were associated with higher intensity of low back pain; the greater LLD the greater the intensity [17]. The current patient had an LLD of 3.8 cm (1.5 inches) confirmed by X-ray. This level of discrepancy alters the normal biomechanics and results in functional limitations, including posture and gait abnormalities [18].

LLDs are associated with several musculoskeletal disorders, including scoliosis and resultant degenerative spinal changes. LLD has been shown to cause pelvic obliquity in the frontal plane. To maintain shoulder balance and compensate for the pelvic obliquity, Cumming *et al*. [19] noted that functional lumbar scoliosis occurs in LLD patients, with convexity directed toward the shorter lower limb [4].

Busquet’s theory can help elucidate the potential compensatory role of abdominal muscles in LLD.

Busquet states that the compensatory mechanism of anatomical LLD involves the anterior movement of the iliac bone, with a rebalance of the contralateral hemipelvis using the extension muscle chain to lengthen the shorter lower limb, combined with a posterior position of the contralateral iliac bone on the longer lower limb side. This, in turn, stimulates the flexion muscle chain, including the RA and hamstring muscles, which led, as in this case, to the first event of compensation: chronic flexion of the trunk [20, 21].

With an asymmetric tilt of the pelvis to the left, a second compensation occurs over time, generating pelvic torsion and, therefore, contralateral trunk torsion with left EO contraction [19, 20]. The same kind of compensation appears in patients with lower extremity amputations who exhibit a greater thickness of the EO and IO on the amputated side [10].

A chronically tonic-contracted muscle, as seen in the current patient, may result in hypertrophy and then ischemia, with a subsequent drop in pH. This, in turn, stimulates the vanilloid receptor (VR-1), which is sensitive to low pH, and the purinergic receptors, which are activated by adenosine triphosphate (ATP) released by damaged cells. This cascade may explain why pain is felt. This same nociceptive mechanism has been described for bruxism [22].

The most frequent lower limb compensation in both a static position and during gait is hip extension, with knee and plantar flexion of the ankle in the shorter lower limb and flexion of the hip and knee in the longer lower limb [17]. Together, these constrain the longer lower limb’s hip, creating a greater need for hip arthroplasty [23]. However, the current patient had an osteoid osteoma in his left hip, which explains the degeneration seen in the shorter lower limb. Abdominal pain is a common complaint [24].

In many cases it can progress to chronic pain, which is difficult to investigate and treat [25]. However, despite their essential role in pelvic balance, spine stasis, and trunk stability, the abdominal muscles have not been thoroughly investigated [25]. Moreover, they are often underexamined and largely ignored in the literature when it comes to LLD [26]. Regarding the current patient’s treatment, his posture was rebalanced to a 1.5 cm (0.6 inches) residual inequality using a corrective heel raise, which is indicated for LLD < 5 cm (2 inches) [16]. Lidocaine and botulinum toxin-A treatment was alternated on hyperactive muscles, which yielded good results, despite the limited evidence regarding their efficacy in the literature [27]. This resulted in a long-term reduction in pain and a resultant reduction in analgesic use [28, 29].

Throughout this therapeutic strategy, there was an almost total disappearance of the pain linked to the patient’s most recent compensatory phenomenon (left rotation), while a slower decrease was observed in the oldest compensatory phenomenon (flexion). However, it is important to note that treatment of this phenomenon began later.

This case report has limitations inherent to its methodological nature. An individual analysis should be made when applying these case findings to other patients. There is a lack of literature on previous cases presenting with a similar clinical context; nevertheless, after careful analysis of the differential diagnosis, we were able to draw conclusions and develop an appropriate therapeutic approach. This case follow-up was optimal, and the measurements performed were sufficiently objective to make it possible to demonstrate clinical improvement.

The treatment of chronic musculoskeletal pain, particularly in patients over 30 years old, is long-term work. Pain relief is an important outcome for patients, especially if it improves their quality of life. Incorrect diagnoses can result in the inadequate management of this condition and therapeutic escalation, which creates a high risk of patient dissatisfaction due to a lack of efficacy and to adverse effects [29].

As explained above, muscle pain is caused by contractures related to posture compensation. Although botulinum toxin has a controversial action in the treatment of musculoskeletal pain [30], its use was justified for this current case due to the specific context of pain related to muscle contracture [31, 32]. Muscle hyperactivity was highlighted via electromyography (auditory feedback alone).

It is critical to not trivialize the clinical examination and anamnesis of patients who have been examined many times previously. New information may be found, or previously ignored information may be properly considered and understood. Particularly multifocal pain requires precise analysis to determine the different kinds of pain, with separate outcome objectives set in relation to each pain location.

In abdominal pain, the most important aspect of history-taking is to identify any features that do not indicate a functional gastrointestinal disorder.

A biomechanical compensation explained this patient’s symptom and shows us how LLD can cause parietal abdominal pain. An LLD of 2–5 cm can be equalized using a shoe raise and/or insoles.

## Data Availability

The patient signed the written contentment form where he agreed to participation and the publication of his clinical data and photographs with the proper masking. One single patient’s information was recovered for this case report. No metadata was generated or shared.

## Abbreviations

ACNES: Anterior cutaneous nerve entrapment syndrome
ATP: Adenosine triphosphate
CPRD: Truncal complex regional pain syndrome
CT: Computed tomography
DN-4: Neuropathic pain in 4 questions
EO: External oblique muscle
ICF: International Classification of Functioning
IO: Internal oblique muscle
LLD: Lower limb length discrepancy
NRS-11: Numerical rating scale 11
RA: Rectus abdominis muscle
TrA: Transverse abdominal muscle
VR-1: Vanilloid receptor 1
